# Impact of climate and host availability on future distribution of Colorado potato beetle

**DOI:** 10.1038/s41598-017-04607-7

**Published:** 2017-07-03

**Authors:** Cong Wang, David Hawthorne, Yujia Qin, Xubin Pan, Zhihong Li, Shuifang Zhu

**Affiliations:** 1 College of Plant Protection, China Agricultural University, Beijing, 100193 China; 2 Institute of Plant Quarantine, Chinese Academy of Inspection and Quarantine, Beijing, 100029 China; 3 Department of Entomology, University of Maryland, 4112 Plant Science Building, College Park, Maryland 20742 USA

## Abstract

Colorado Potato Beetle (CPB) is a devastating invasive pest of potato both in its native North America and now across Eurasia. It also damages eggplant, tomato and feeds on several wild species in the Solanaceae, such as *S*. *eleagnifolium* and *S*. *rostratum* Dunal (SR). Since first categorized as a pest in 1864, CPB has spread rapidly across North America, Europe and Asia. In light of its invasiveness and economic importance, it is necessary to study how climate change and host availability may alter the distribution of the CPB. Maximum Entropy (MaxEnt) models were used to anticipate global range expansion as influenced by environmental conditions, and by the possibility of cooperative invasion of CPB and its wild host SR. The results indicate that both CPB and SR can occupy warm areas of North America, South Africa, Europe, China, and Australia. Future climate conditions may promote CPB expansion into northern regions and SR into the circumpolar latitudes. The existing range and continued spread of SR may also assist the global expansion of CPB. Future management of this pest should consider the impacts of global climate change and host availability on its potential global distribution.

## Introduction

The Colorado Potato Beetle (CPB), *Leptinotarsa decemlineata* (Say) (Coleoptera: Chrysomelidate), is a devastating potato pest. CPB was first discovered in North America in 1811, feeding on *Solanum rostratum* Dunal (SR)^1^. After it was first observed damaging potato crops in the 1850s, it rapidly spread, infesting potato fields across most of the United States and southern Canada by 1875^1^. By the 1950s, CPB spread to Europe and Central Asia, moving eastward, through Eastern Europe, Russia and Kazakhstan, finally reaching China in the 1990 s^2–4^. Where it occurs, CPB is the most destructive insect pest of potato (*S*. *tuberosum*) and frequently damages eggplant (*S*. *melangena*) and tomato (*Lycopersicon esculentum*)^5, 6^. The beetle can cause 20–100% reductions in potato yield through consumption of foliage^7^ and directly damages eggplant and tomato by feeding on young fruits^8^. Therefore, it is necessary to identify current and future areas worldwide that may be suitable to this pest, including areas not yet colonized, to develop measures for prevention of CPB colonization and its damaging consequences^9^.

By natural pathways, it may take CPB decades to disperse into other potato growing regions. For example, in China, CPB first entered from Kazakhstan, and was recorded on potato crops in the Ili River Valley of Xinjian province in 1993^10^. Since then, it has been found in an area of 260,000 hectares^11^. It is anticipated that with the aid of favorable winds, this insect could spread, unaided by humans, 100–200 km/year into suitable habitat^12^. CPB invasion of China is of considerable concern because it threatens a rapidly growing potato industry though it is not yet found in most potato cultivation regions of China. The dispersal dynamics of this pest may be altered by the effects of climate change on both the insect and its host plants. Thus, the pest risk analysis must take the impact of future climate change into consideration.

Climate change can also influence the distribution of CPB by changing the abundance and distribution of its cultivated and uncultivated host plants. The distribution areas of crop host plants may be influenced by climate change, as observed for CPB and potato in Europe^13^. A key non-crop host plant of CPB, *Solanum rostratum* (SR) is also a globally invasive weed^14^. SR is a member of the Solanaceae and a perennial herbaceous plant. It is native to Mexico and the southwestern United States, but is now found throughout North America, Europe, Asia and Australia^14, 15^. In this case, the distribution of two different invasive species, a weed and an herbivore, can interact during invasions^16^. Climate change may influence invasion pathways of CPB by increasing the distribution and abundance of this weed. It is therefore necessary to study the current and future climate change-mediated global distributions of both species.

Climate change and host availability can affect the distribution of insect invasions on a global scale. Berzitis *et al*.^17^ has found that climate change and host plant availability may influence the expansion of suitable areas for the bean leaf beetle (*Cerotoma trifurcata*) over time. Bacon *et al*.^18^ also indicated that three factors: propagule pressure, climate suitability and host availability, can explain insect invasions in Europe. Because propagule pressure only had a positive effect when considered together with climate and host, and previous studies have indicated that CPB is quite sensitive to the climate, it is a good candidate for range expansion due to climate change^19, 20^.

From 1990s to 2010s, a number of studies have used species distribution models to assess the potential distribution of CPB^9, 21–25^. Rafoss and Sæthre^22^ used CLIMEX to analyze the spatial and temporal distribution of CPB in Norway, with the result that the pest might be able to establish in eastern Norway. Similar studies have also been conducted in the Czech Republic, UK and Europe^24, 25^. Additional information expanding the known current distribution of CPB has subsequently been gathered that could alter estimates of this specie’s suitable distribution. Because non-cultivated host plants, and SR in particular, serve as conduits for range expansion of this pest insect, we also include analysis of this plant, including changes in its distributional range that may occur due to climate change. Analysis of current and potential future distributions of this weed will improve our ability to understand the risks of CPB invasion. In this study, we used the Maximum Entropy (MaxEnt) model to estimate the future climate change impact on CPB and SR and their potential invasion ranges at a global scale to provide the basis for their prevention and control.

## Results

PCA analysis of 19 climate-related variables revealed differences between CPB and SR in the principal components for variable selection. For CPB, the first four principal components explained 93% of the total variance with the first component mainly attributed to precipitation during wet and cold seasons, the second attributed to temperature in cold and dry periods, the third to temperature in warm periods and the mean temperature of the warmest month, and the fourth to the seasonality of precipitation and the precipitation in the dry periods. For SR, the first five principal components accounted for 94% of variation with the first component composed of seven variables related to temperature, the second attributed to the seasonality of precipitation and precipitation in the dry periods, the third to temperature in warm periods, the fourth to precipitation in the wet periods, and the fifth to temperature in the wet periods and precipitation in the cold periods. Table 1 shows PCA scores of all variables for CPB and SR, and those variables used in the model.

**Table1:** Principal component analysis (PCA) performed on 19 bioclimatic variables for Colorado potato beetle (CPB) and *S*. *rostratum Dunal* (SR), which was carried out in IBM SPSS Statistics version 22 (https://www.ibm.com/support/docview.wss?uid=swg21646821).

Bioclimatic variables	Principal Components for CPB				Principal Components for SR				
	1	2	3	4	1	2	3	4	5
Annual mean temperature (bio1)^#^	0.29	0.683	0.663	0.008	0.725	0.205	0.622	0.058	0.158
Mean diurnal range (bio2)*	0.015	−0.123	0.808	−0.345	0.084	0.643	0.597	0.007	0.110
Isothermality (bio3)^#^	0.574	0.607	0.43	−0.144	0.734	0.513	0.121	0.099	0.058
Temperature seasonality (bio4)^#^*	−0.354	−0.924	0.042	0.015	−0.865	−0.135	0.461	−0.036	0.050
Max temperature of warmest month (bio5)^#^	0.091	0.111	0.98	−0.047	−0.006	0.304	0.946	−0.012	0.055
Min temperature of coldest month (bio6)^#^*	0.334	0.899	0.262	0.084	0.977	0.004	−0.067	−0.008	−0.111
Temperature annual range (bio7)^#^	−0.302	−0.893	0.299	−0.117	−0.703	0.194	0.661	−0.002	0.116
Mean temperature of wettest quarter (bio8)^#^	0.173	0.173	0.793	−0.099	−0.026	0.177	0.563	0.110	0.727
Mean temperature of driest quarter (bio9)^#^*	0.249	0.809	0.45	0.065	0.767	0.198	0.162	−0.083	−0.486
Mean temperature of warmest quarter (bio10)^#^*	0.122	0.232	0.945	0.04	0.013	0.095	0.962	0.006	0.158
Mean temperature of coldest quarter (bio11)^#^	0.337	0.846	0.41	0.006	0.961	0.217	0.145	0.043	0.040
Annual precipitation (bio12)^#^*	0.887	0.365	0.152	0.183	−0.104	−0.528	−0.048	0.817	−0.109
Precipitation of wettest month (bio13)*	0.851	0.41	0.262	−0.073	0.100	0.122	0.043	0.977	0.008
Precipitation of driest month (bio14)	0.619	0.15	−0.104	0.727	−0.139	−0.931	−0.172	0.145	−0.044
Precipitation seasonality (bio15)^#^*	0.22	0.167	0.541	−0.75	0.259	0.843	0.201	0.321	0.071
Precipitation of wettest quarter (bio16)^#^	0.867	0.407	0.225	−0.064	0.086	0.075	−0.007	0.985	−0.051
Precipitation of driest quarter (bio17)^#^*	0.648	0.183	−0.037	0.711	−0.137	−0.933	−0.146	0.183	−0.072
Precipitation of warmest quarter (bio18)	0.886	0.227	0.085	0.037	−0.213	−0.269	0.085	0.625	0.644
Precipitation of coldest quarter (bio19)^#^*	0.834	0.286	0.1	0.331	0.070	−0.320	−0.144	0.351	−0.827

^*^10 bioclimatic variables used in the analysis of CPB; ^#^15 Bioclimatic variables used in the analysis of SR. The components were scaled between 0–1; the closer the values to one, the more variance they explain.

In support of the model performance, the AUC values of CPB and SR were 0.960 and 0.934 respectively, indicating that the MaxEnt models accurately discriminated between the suitable and unsuitable areas for CPB and SR (see Supplementary Fig. S1). The *p*-*values* of Cumulative Binomial Probability Distributions Test for CPB and SR were both less than 0.01, and the probabilities of successfully predicted test data of CPB and SR were 0.752 and 0.853, indicating a high proportion of correctly predicted test occurrences in our modelling.

For CPB models, the jackknife test indicate that the climate variables with highest gain when used in isolation were Bio6 (Min Temperature of Coldest Month) and Bio9 (Mean Temperature of Driest Quarter). Additionally, Bio10 (Mean Temperature of Warmest Quarter) was also found to influence the distribution of CPB (see Supplementary Fig. S2). Bio1 (Annual Mean Temperature), Bio11 (Mean Temperature of Coldest Quarter), Bio6 (Min Temperature of Coldest Month) and Bio9 (Mean Temperature of Driest Quarter) influenced the model results of SR when used in isolation (see Supplementary Fig. S2). MaxEnt’s default analysis of variables contributions showed the percent predictive contribution of each used climate variable. The higher the contribution, the more impact that particular variable has on predicting the occurrence of that species. For CPB, Bio6 (Min Temperature of Coldest Month) had the highest contribution of 24.7%. For SR, Bio1 (Annual Mean Temperature) had the highest contribution of 53.1%.

**Figure 1:**
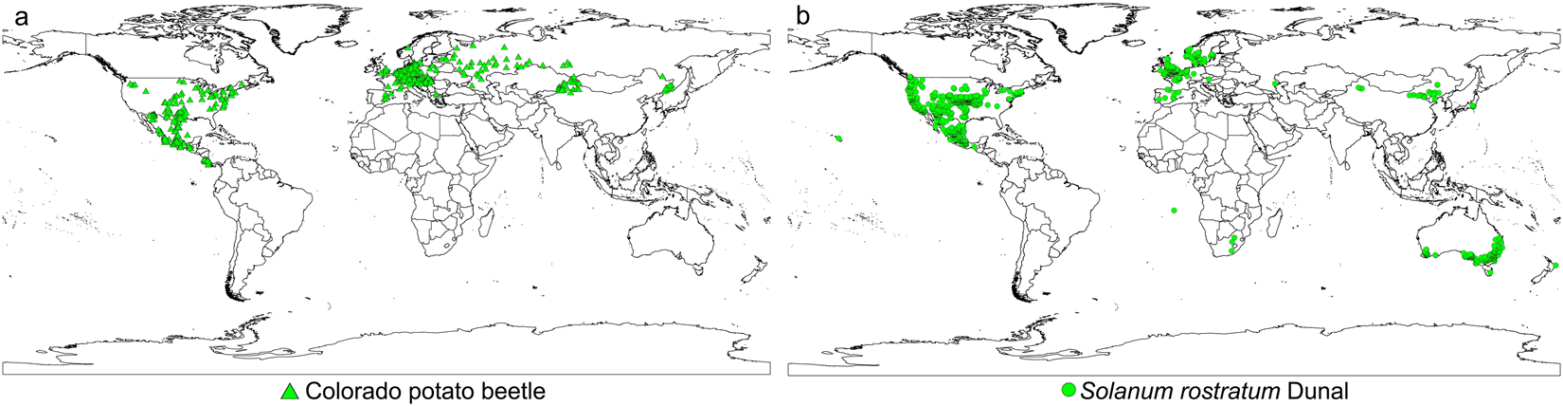
Global distribution data used to build and evaluate the MaxEnt models of two species. (**a**) 670 global distribution points of Colorado potato beetle (CPB); (**b**) 1090 global distribution points of *S*. *rostratum* Dunal (SR). Both maps are generated by using the tool of ArcGIS 10.2.2 (ESRI, Redlands, CA, USA, http://www.esri.com/).

**Figure 2:**
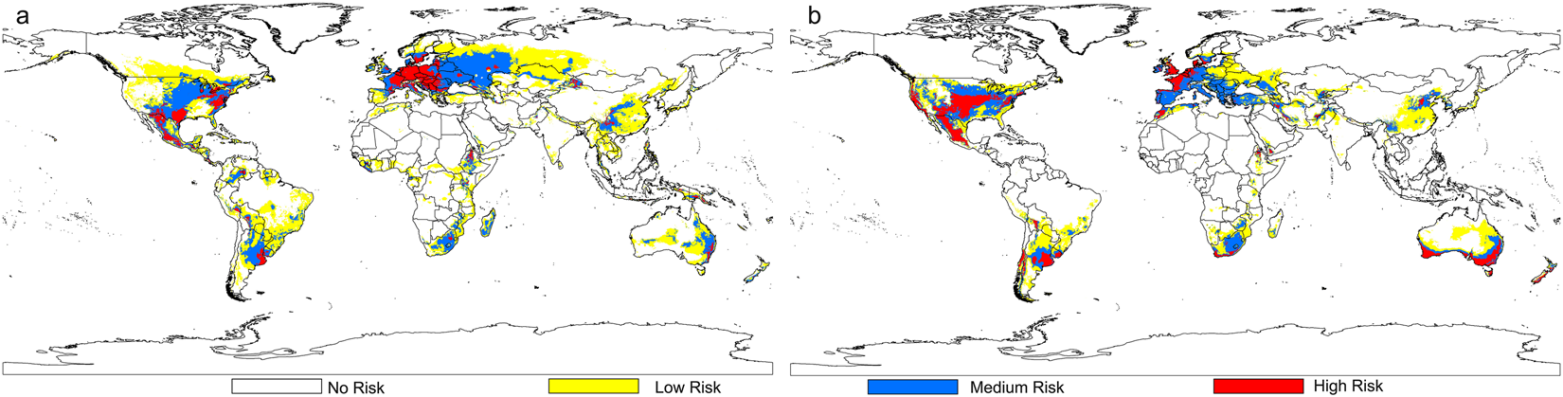
Potential global distribution maps for Colorado potato beetle (CPB) and *S*. *rostratum* Dunal (SR), which were produced by MaxEnt (v3.3.3k, http://biodiversityinformatics.amnh.org/open_source/maxent/) under current climate conditions. (**a**) Habitat suitability of CPB; (**b**) habitat suitability of SR. White color represents no risk areas, yellow color represents low risk areas, blue color represents medium risk areas and red color represents the high risk areas. The whole maps are generated by using the tool of ArcGIS 10.2.2(ESRI, Redlands, CA, USA, http://www.esri.com/).

**Figure 3:**
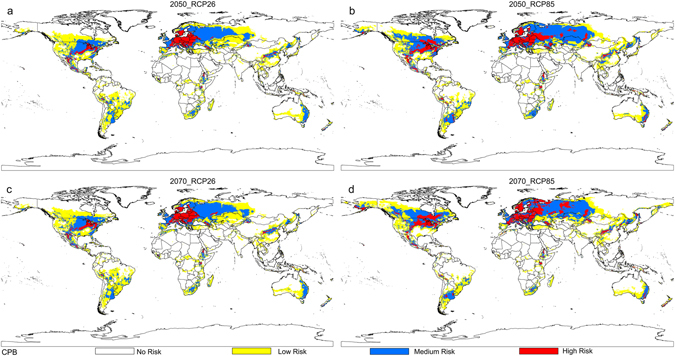
Potential global distribution maps for Colorado potato beetle under future climate conditions, which were produced by MaxEnt (v3.3.3k, http://biodiversityinformatics.amnh.org/open_source/maxent/). (**a**) Mean predicted result for four global climate models (GCMS): IPSL-CM5A-LR (IP), NorESM1-M (NO), HadGEM2-ES (HE) and MIROC-ESM-CHEM (MI), which was modeled under 2050-RCP26; (**b**) mean predicted result for four GCMS, which was modeled under 2050-RCP85; (**c**) mean predicted result for four GCMS, which was modeled under 2070-RCP26; (**d**) mean predicted result for four GCMS, which was modeled under 2070-RCP85. White color represents no risk areas, yellow color represents low risk areas, blue color represents medium risk areas and red color represents the high risk areas. The whole maps are generated by using the tool of ArcGIS 10.2.2(ESRI, Redlands, CA, USA, http://www.esri.com/).

**Figure 4:**
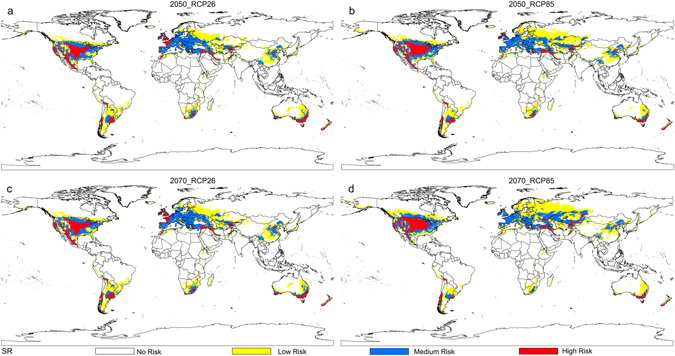
Potential global distribution maps for *S*. *rostratum* Dunal under future climate conditions, which were produced by MaxEnt (v3.3.3k, http://biodiversityinformatics.amnh.org/open_source/maxent/). (**a**) Mean predicted result for four global climate models (GCMS): IPSL-CM5A-LR (IP), NorESM1-M (NO), HadGEM2-ES (HE) and MIROC-ESM-CHEM (MI), which was modeled under 2050-RCP26; (**b**) mean predicted result for four GCMS, which was modeled under 2050-RCP85; (**c**) mean predicted result for four GCMS, which was modeled under 2070-RCP26; (**d**) mean predicted result for four GCMS, which was modeled under 2070-RCP85. White color represents no risk areas, yellow color represents low risk areas, blue color represents medium risk areas and red color represents the high risk areas. The whole maps are generated by using the tool of ArcGIS 10.2.2(ESRI, Redlands, CA, USA, http://www.esri.com/).

Based on the distribution data of three databases, collection efforts and literature, the current distribution maps of CPB and SR were shown in Fig. 1, and their habitat suitability was shown as different colors on the model maps (Figs 2, 3 and 4). The averaged value of 10 percentile training presence logistic threshold for CPB was 0.163 and for SR was 0.195. Based on the averaged threshold value, we categorized habitat suitability of CPB and SR into 4 levels: no risk (0.00–0.17), low risk (0.17–0.42), medium risk (0.42–0.67), and high risk (0.67–1.00).

MaxEnt’s estimation of the global distribution of CPB under current climate conditions indicated that highly suitable regions include the southeast of North America, central and western regions of South America, central and western regions of Europe, eastern regions of Africa and Asia, and eastern regions of Australia and New Zealand (Fig. 2a). As the native range of CPB includes most of southern and central North America (Mexico and the United States), it was reassuring that MaxEnt identified those areas as suitable for its survival. Similarly, the contemporary range of this insect now includes most of Europe and the MaxEnt model also accurately indicated that regions eastward, including the north-western provinces China and Europe, are climatically suitable for CPB. Of considerable interest were those regions not currently home to CPB which were found to have suitable climactic conditions for the pest, including large portions of central and south America, substantial areas in northern, central and southern Africa and Asia, including Madagascar, Asia Minor, Pakistan, India, Bangladesh, Nepal, much of eastern China and large areas in Australia (Fig. 2a).

A similar map, using contemporary conditions, was developed for SR, which is native to Mexico and the south-central United States and is found throughout the U. S. (Fig. 2b). The contemporary distribution of SR under current climate conditions was similar to that of CPB, although southern Africa and Australia were found to be more suitable for SR than for CPB. Our MaxEnt results also correctly simulated the overlapping distributions of CPB and SR (Fig. 2).

Four distribution maps of suitable habitat for CPB under a range of possible future climate scenarios for 2050 and 2070 are summarized in Fig. 3. This figure shows mean predicted results of all four climate change models under two RCPs (RCP26 and RCP85), which represent the highest and lowest of the four greenhouse gas concentration pathways. Most areas that are currently suitable for CPB will remain so into the 2050 s and 2070 s under these climate change scenarios. This can be seen across western Europe, eastern Australia, central China and southern Russia. However, areas projected suitable for CPB in Africa contracted under the future climate scenarios. The 2050 and 2070 climate projections may result in CPB range expansion into key areas of concern, including China, Canada, and Russia.

Figure 4 shows the effect of climate scenarios on the potential future invasive distributions for SR. Suitable areas for SR are projected to expand when comparing RCP26 to RCP85 from 2050 s to the 2070 s, with suitable habitat projected to shift polewards for both species. Areas with low and moderate suitability for SR under current climate conditions are projected to expand with the changing RCPs, especially in central Europe. Central America may become a high risk area for SR invasion by the 2070 s under RCP 85 (Fig. 4d).

The distribution maps of CPB and SR, show large areas of overlap in suitable habitat for both species, now and in the future. For example, Western Europe, eastern Australia, central China, southern America and southern Russia are likely to be suitable for both species simultaneously.

**Figure 5:**
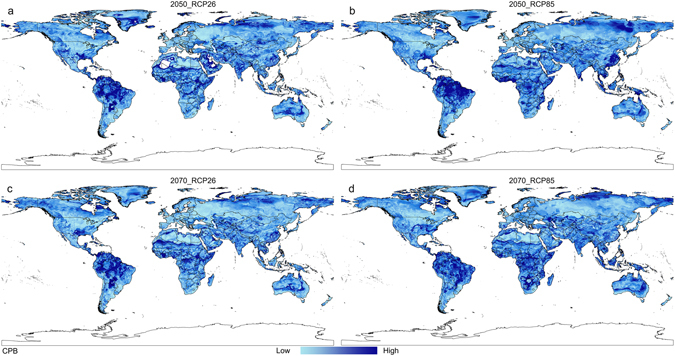
Coefficient of variation (CV) maps of four global climate models (GCMS) for Colorado potato beetle which were produced by MaxEnt (v3.3.3k, http://biodiversityinformatics.amnh.org/open_source/maxent/) under future climate conditions. (**a**) Variance among four GCMS under 2050-RCP26; (**b**) variance among four GCMS under 2050-RCP85; (**c**) variance among four GCMS under 2070-RCP26; (**d**) variance among four GCMS under 2070-RCP85. High values (warmer colors areas) indicate higher uncertainty with respect to low values (colder colors areas) where different models produce similar results. The whole maps are generated by using the tool of ArcGIS 10.2.2(ESRI, Redlands, CA, USA, http://www.esri.com/).

**Figure 6:**
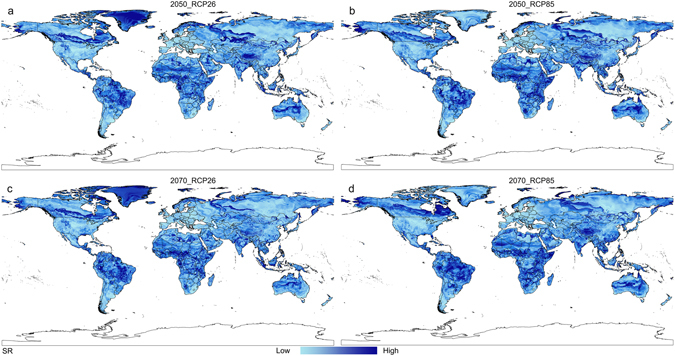
Coefficient of variation (CV) maps of four global climate models (GCMS) for *S*. *rostratum* Dunal which were produced by MaxEnt (v3.3.3k, http://biodiversityinformatics.amnh.org/open_source/maxent/) under future climate conditions. (**a**) Variance among four GCMS under 2050-RCP26; (**b**) variance among four GCMS under 2050-RCP85; (**c**) variance among four GCMS under 2070-RCP26; (**d**) variance among four GCMS under 2070-RCP85. High values (warmer colors areas) indicate higher uncertainty with respect to low values (colder colors areas) where different models produce similar results. The whole maps are generated by using the tool of ArcGIS 10.2.2(ESRI, Redlands, CA, USA, http://www.esri.com/).

Figures 5 and 6 are the CV maps of CPB and SR model predictions. Variation between scenarios of CPB models was mainly concentrated on northern South America, some eastern parts of Russia, as well as some limited regions of Africa and Asia (Fig. 5). As for SR, variation in suitable habitat across the various climate scenarios was mainly concentrated in northern America, and limited regions of Africa, Asia and Australia (Fig. 6). Generally, however, there was agreement in the distribution of suitable habitat under the various climate scenarios.

## Discussion

Our models indicate that the global distribution of suitable habitat for CPB will be substantially affected by climate change. Generally, suitable habitat in the northern hemisphere is projected to expand northward as climate changes and these areas will become more climatically suitable. Most of the influential variables in the CPB models have a strong connection with temperature, resulting in significantly increased global distributions of CPB with increased future temperatures. Numerous studies on the potential effect of climate change on CPB distribution have been motivated by the temperature dependence of CPB, especially the coldest temperatures that allow diapause of CPB in winter^20, 25–27^. Long-term risks are, therefore, greater in higher latitude areas, since these regions will experience warming that will facilitate CPB invasion and establishment.

Comparison of simulation results of CPB and SR under different climate scenarios indicates that areas projected to be suitable coincide in spatial extent. This is important because SR may serve as a food resource during colonization of new areas or in established regions during seasons that are lacking potato or other Solanaceae crops^15, 16^. Because SR is not the only wild host plant of this insect^1^ we suggest that understanding of the current and future distribution(s) of additional wild hosts, would further aid our assessment of risks of CPB invasion.

A previous study used the mechanistic model CLIMEX to predicted suitable habitat for CPB^21^. These study did not concluded that Kazakhstan, southern Russia and northwest China would not be suitable for this pest, but all have since been colonized by CPB^11, 28^. In a previous study^29^, we also used CLIMEX to assess the potential distribution of suitable habitat of CPB in Kazakhstan, southern Russia and northwest China and achieved results similar to those of MaxEnt. In this research, the selection of MaxEnt can avoid potential errors that may occur when physiological data (used in CLIMEX) is uncertain or inaccurate^30^. MaxEnt may also perform better than other correlative models when the true absence data are unavailable^31, 32^. But it still has limitations, such as it requires enough present points to have reliable modelling performance^33, 34^. Thus utilizing more than one species distribution models may reduce model-related uncertainty and improve the prediction capacity.

In addition to the limitation of including only a single wild host plant of CPB, several uncertainties remain in modelling future habitat for CPB. Spatial bias in occurrence records can reduce the quality of distribution models^33, 35, 36^. Although we have undertaken several measures to reduce spatial bias, some causes, such as the sample selection bias caused by differing sample collection conditions and recordings across the whole world^36^, remains a concern. These biases are difficult to remedy and should be taken into consideration when using all of the models’ results^33^. Moreover, CV maps indicate the climate scenarios used to develop our distribution maps vary, and this may create uncertainty in the CPB’s future distribution^17, 37, 38^. Further, additional human-mediated changes to the agricultural environment, such as irrigation or greenhouses, may lead to range expansion of CPB into regions not otherwise suitable for it^39, 40^. CPB is also capable of adapting to novel conditions, potentially expanding its fundamental range beyond current or historical limits^37^. Additional research on the evolution of expanded environmental limits for this species will be valuable for future versions of these models.

AUC and Cumulative Binomial Probability Distributions Test have been used as measures of our model quality. Both indicated that the performance of MaxEnt model is acceptable. We also compared predicted ranges with published ranges, providing additional confirmation of the predictive power of our models. However, the AUC in MaxEnt does not represent the “true” AUC (as the default setting of MaxEnt was used to calculate AUC values, the background data may or may not be true absences) and can be overestimated^41–43^.

Our results update previous studies by taking global climate change and host availability into account. As a global-scale study, our research can contribute to pest risk analysis and inform plant quarantine policy. Here we show that this beetle has not reached its maximum global geographic range but its spread has slowed considerably in recent years, partly because of the international collaborative action^5, 44, 45^. These distribution maps of CPB and its hosts can be used to design more detailed future surveys and support better planning for quarantine and control measures.

## Materials and Methods

### Species distribution data

The global occurrence data of CPB and SR were extracted mainly from three databases: the Global Biodiversity Information Facility (GBIF, http://www.gbif.org), the CABI Crop Protection Compendium (CABI CPC, http://www.cabi.org/cpc) and the Plant Quarantine data retrieval system (PQR-EPPO, http://www.eppo.int). However, for CPB, these sources yielded very few records from Asia, especially from China and Russia. Hence, we included data from additional collections throughout China, USA and Mexico which were conducted by our research group in the field. And we also used the Russian distribution data of CPB from Popova^28^. Data from both native and invaded ranges of CPB and SR was used for model calibration. CPB distribution data dated from 1896 to 2016, and for SR from 1948 to 2016. Before use, all of the data were compared and manually checked for accuracy. Entries with missing, duplicate or clearly false locations were deleted. We also assigned the distribution data of CPB and SR to a coarse resolution (0.5° × 0.5°) by using the workflow of Li *et al*.^46^ to minimize bias which is a sampling bias or error at a fine resolution in these three databases. The total data set contained 670 records for CPB and 1090 records for SR at 0.5° × 0.5° resolution (Fig. 1), and all the conversion were conducted in ArcGIS 10.2.2 (ESRI, Redlands, CA, USA, http://www.esri.com/).

### Climate Variables

Climate data were obtained from the WorldClim database (http://www.worldclim.org/). Current climate conditions were represented by monthly average data from 1950–2000. Future climate conditions, estimated for 2050 (average for 2041–2060) and 2070 (average for 2061–2080), were derived from four global climate models (GCMS): IPSL-CM5A-LR (IP), NorESM1-M (NO), HadGEM2-ES (HE) and MIROC-ESM-CHEM (MI), which belong to the most recent GCM climate projections that are used in the Fifth Assessment Intergovernmental Panel on Climate Change (IPCC) report (http://www.ipcc.ch/report/ar5/). These models were selected to give a wide range of rainfall and temperature changes, rather than to represent the likelihood of future climate change^47^. The models were each run using four representative concentration pathways (RCPs) that differ in their greenhouse gas concentrations - RCP26, RCP45, RCP60 and RCP85 from the Fifth Assessment Report of IPCC 2014^48^.

The bioclimatic variables are those often used in species distribution modeling. They were derived from monthly temperature and rainfall values in order to generate more biologically meaningful variables. We included all 19 bioclimatic variables (Table 1) that may influence the survival and establishment of the invasive insect and its host plant. Because some of these variables are highly correlated, principal component analysis (PCA) was performed to assist selection of a set of uncorrelated variables that are useful and eco-physiologically relevant. All climate variables were at a spatial resolution of 5 arc-min (ca. 9 km at the equator). They have been modified and extracted in Spatial Analyst function of ArcGIS version 10.2.2 (ESRI, Redlands, CA, USA, http://www.esri.com/) to be the same spatial resolution (0.5° × 0.5°) as the species distribution data. The PCA analysis were carried out in IBM SPSS Statistics version 22 (https://www.ibm.com/support/docview.wss?uid=swg21646821).

### MaxEnt Modeling

MaxEnt was employed to simulate changes in the distribution of suitable habitat^31^. It estimates suitable habitat of species by integrating detailed climate variables with species’ current locations^49–52^. MaxEnt was developed to use presence-only data, which is the type of species distribution data available for CPB and SR.

Projections of the geographic distributions of CPB and SR were inferred with MaxEnt (v3.3.3k, http://biodiversityinformatics.amnh.org/open_source/maxent/). Models were calibrated using 75% of the available records for each species as training data, and the remaining 25% were used for model validation as testing data. MaxEnt was run with the default convergence threshold (10^−5^), the maximum number of iterations (5000) and default features. As no absence background data were available for CPB and SR, we used the minimum convex polygon to define the background area (around the CPB and SR presence areas) in ArcGIS 10.2.2 (ESRI, Redlands, CA, USA, http://www.esri.com/). Ten thousand global background points were used to run the models. This allows models to have adequate time for convergence. Models were each run five times to measure internal model variability. The logistic format of MaxEnt output was used in our research to estimate the probability of presence (range from 0 to 1). The minimum probability of suitable habitat in MaxEnt was set to “10 percentile training presence logistic threshold”. We calculated the averaged value of threshold in the simulation process. This value was used to denote no risk habitat: if the suitability value was greater than the threshold, it was be considered as risk habitat. To maximize the predictive information and simplification of future analysis future analysis, we depicted habitat suitability at 4 levels: no risk, low risk, medium risk and high risk. All detailed information of MaxEnt workflow (including data processing) could be seen in Young *et al*.^53^. Results and variability of replicated model runs were converted into raster files and calculated in ArcGIS 10.2.2 (ESRI, Redlands, CA, USA, http://www.esri.com/). In order to map the variation among four global climate change models, the coefficient of variation (CV) between different scenarios was calculated and mapped^54, 55^.

The Jackknife test was used to measure each variable’s importance in model development. The relative importance of a variable was evaluated using default estimates of percentage model contribution^46^. Receiver operating characteristic (ROC) analysis was used to evaluate the fit of the model to the data and model performance and the area under ROC curve (AUC) was used as an index to provide overall accuracy estimates^32, 50–52^. AUC ranges between 0 and 1, and models with an AUC value higher than 0.75 are considered acceptable^56^. To calculate the proportion of correctly predicted test occurrences, we calculated the cumulative binomial probability distributions. Using the test data prediction results as measures of success and the proportion of the area predicted to be suitable as a null expectation of the probability of success, and the total number of occurrences as the number of trials, *p*-*values* have been obtained by using R-3.3.3 (https://cran.r-project.org/)^57^.

### Data Availability

The datasets generated during and analyzed during the current study are available from the corresponding author on reasonable request.

## Electronic supplementary material

Supplementary Information
