# Supplementary material for: Impact of climate and host availability on future distribution of Colorado potato beetle
Source: Sci Rep. 2017 Jul 3;7:4489. doi: 10.1038/s41598-017-04607-7 (PMC5495769; doi:10.1038/s41598-017-04607-7)
Supplement: Supplementary file 1 — Supplementary Information [file 41598_2017_4607_MOESM1_ESM.pdf]

Impact of climate and host availability on future distribution of Colorado potato beetle

CongWang[1, 2], David Hawthorne[3], Yujia Qin[1], Xubin Pan[2]*, Zhihong Li[1]*, Shuifang Zhu[2]*

1. College of Plant Protection, China Agricultural University, Beijing 100193, China

2. Institute of Plant Quarantine, Chinese Academy of Inspection and Quarantine, Beijing 100029, China

3. Department of Entomology, University of Maryland, 4112 Plant Science Building, College Park, Maryland 20742, USA

* Correspondence and request for materials should be addressed to:

X.P. (xubin.hu.pan@gmail.com) or Z.L. (lizh@cau.edu.cn) or S.Z. (zhusf@caiq.gov.cn)

China Agricultural University and Chinese Academy of Inspection and Quarantine have contributed equally to this work.

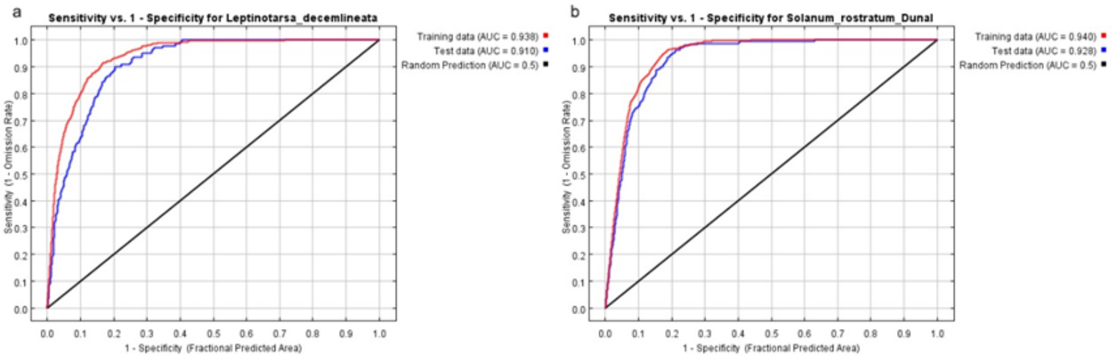

Supplement Figure1. The receiver operating characteristics curve (ROC) for training data of both species with the area under the ROC curve (AUC). (a) ROC and AUC of Colorado potato beetle; (b) ROC and AUC of *Solanum rostratum* Dunal. These pictures were produced by Maxent (v3.3.3k, http://biodiversityinformatics.amnh.org/open_source/maxent/).

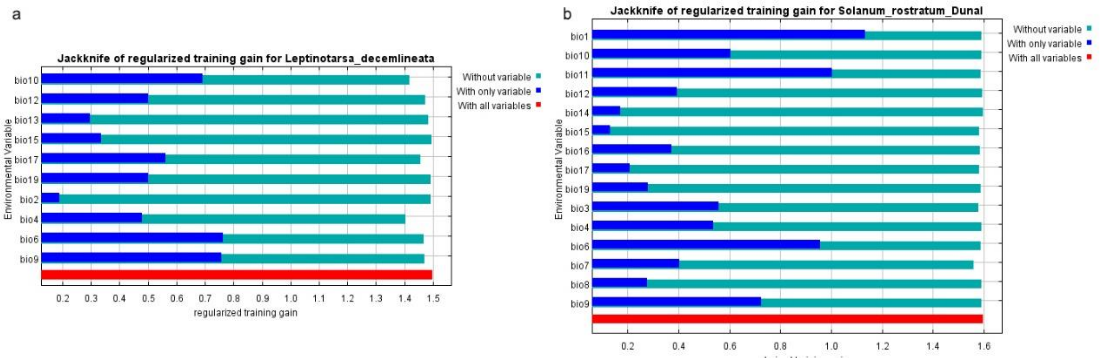

Supplement Figure 2. Jackknife test for relative importance of environmental variables in the development of MaxEnt models. (a) results of 10 environmental variables for Colorado potato beetle; (b) results of 15 environmental variables for *Solanum rostratum* Dunal. These pictures were produced by Maxent (v3.3.3k, http://biodiversityinformatics.amnh.org/open_source/maxent/).
